# Gilteritinib induces PUMA‐dependent apoptotic cell death via AKT/GSK‐3β/NF‐κB pathway in colorectal cancer cells

**DOI:** 10.1111/jcmm.14913

**Published:** 2019-12-27

**Authors:** Liangjun Li, Lin Lin, Ming Li, Weiling Li

**Affiliations:** ^1^ Department of Clinical Laboratory The Second Affiliated Hospital of Dalian Medical University Dalian China; ^2^ Department of Clinical Laboratory The First Affiliated Hospital of Dalian Medical University Dalian China; ^3^ Department of Microecology College of Basic Medical Science Dalian Medical University Dalian China; ^4^ Biotechnology Department College of Basic Medical Science Dalian Medical University Dalian China

**Keywords:** AKT/GSK‐3β/NF‐κB, apoptosis, colorectal cancer, gilteritinib, PUMA

## Abstract

As a highly potent and highly selective oral inhibitor of FLT3/AXL, gilteritinib showed activity against FLT3D835 and FLT3‐ITD mutations in pre‐clinical testing, although its role on colorectal cancer (CRC) cells is not yet fully elucidated. We examined the activity of gilteritinib in suppressing growth of CRC and its enhancing effect on other drugs used in chemotherapy. In this study, we observed that, regardless of p53 status, treatment using gilteritinib induces PUMA in CRC cells via the NF‐κB pathway after inhibition of AKT and activation of glycogen synthase kinase 3β (GSK‐3β). PUMA was observed to be vital for apoptosis in CRC cells through treatment of gilteritinib. Moreover, enhancing induction of PUMA through different pathways could mediate chemosensitization by using gilteritinib. Furthermore, PUMA deficiency revoked the antitumour role of gilteritinib in vivo. Thus, our results indicate that PUMA mediates the antitumour activity of gilteritinib in CRC cells. These observations are critical for the therapeutic role of gilteritinib in CRC.

## 1 INTRODUCTION

In the United States, nearly one‐third of mortality due to cancer are caused by colorectal cancer (CRC).^1^ Colorectal tumours that are metastatic or recurrent cannot be generally cured and have a median overall survival of nearly two years.^1^, ^2^, ^3^ For the treatment for CRC, chemotherapy cytotoxic drugs, such as oxaliplatin, 5‐fluorouracil (5‐FU) and irinotecan, are used in specific combinations, but are not very efficient and cause major side effects because of limited specificity.^2^, ^4^ With the development of antitumour agents that are specifically targeted, chemotherapy against metastatic CRC has undergone significant improvement.^4^, ^5^ For instance, Cetuximab, an anti‐epidermal growth factor receptor (EGFR) monoclonal antibody, is functional singly, or along with cytotoxic drugs for of the therapy of metastatic CRC.^6^, ^7^, ^8^ Thus, more personalized treatments are expected with the advent of specialized targeted therapy in the field of oncological procedures.

FMS‐like tyrosine kinase 3 (FLT3) is associated with the receptor of platelet‐derived growth factor and c‐Kit, having a key role in regulating early haematopoietic cells.^9^, ^10^ FLT3 belongs to the type III family of receptor tyrosine kinases.^11^ The gene coding for FLT3 is present on chromosome 13.q12.^12^ It is expressed chiefly in dendritic cells and human haematopoietic progenitors and has important function in division, differentiation and survival of the leukaemic cells.^12^, ^13^, ^14^ In patients with acute myeloid leukaemia (AML), FLT3 is frequently mutated and is thus associated with poor prognosis and reduced survival of patients.^15^, ^16^ One of frequently observed mutations is internal tandem duplications (ITD) of the juxtamembrane domain, which cause autophosphorylation and dimerization that are ligand‐independent.^17^ When FLT3‐ITD is expressed in cell line that is factor‐dependent, they result in factor‐independent growth and malignant transformation.^9^, ^18^ A newly discovered dual FLT3 inhibitor, gilteritinib (ASP2215),^19^ remarkably decreases the ability of FLT3‐positive leukaemia cells to colonize.^20^ Gilteritinib reduces the extent of FLT3 phosphorylation and its downstream targets in animal models as well as in cell cultures 21 without any obvious toxicity.^16^ Gilteritinib has been approved by the FDA for refractory or relapsed AML with a mutation in FLT3.^22^

Concurrent with regular chemotherapy agents and targeted therapies, apoptosis plays a vital role in the antitumour activities.^23^ While the apoptosis via the mitochondrial activity is mainly regulated by the proteins of Bcl‐2 family.^24^ The distinct and overlapping signals are responded to BH3‐only family members.^25^ Many such proteins, like PUMA (p53 up‐regulated modulator of apoptosis) and Bim, activate Bax/Bak after neutralizing antiapoptotic members of Bcl‐2 family and thus act as strong inducers of apoptosis.^26^ The function of PUMA in initiating both p53‐independent and ‐dependent apoptosis is critical in various cell types.^27^, ^28^ The transcription of PUMA is activated directly by p53 following DNA damage, and in the absence of its induction, the cancer cells lacking p53 are rendered unresponsive to chemotherapy drugs and radiation.^29^ PUMA gets induced by non‐genotoxic stimuli in a p53‐independent manner, through transcription factors such as forkhead box O3a (FoxO3a), NF‐κB and p73.^30^, ^31^ When induced, PUMA activates apoptosis either by activating the Bax and Bak pro‐apoptotic members directly, leading to malfunctional mitochondria and caspase activation cascade and/or by acting as antagonist to antiapoptotic members of the Bcl‐2 family.^31^

In the current study, we aimed to examine the inherent mechanisms of apoptosis induced by gilteritinib and the role of associated pathways in response to gilteritinib therapy in CRC cells. The results provide valuable insight into the mechanism of gilteritinib and its therapeutic responses, a logical basis for manipulating PUMA in enhancing the efficacy of targeted therapies and assessing changes in their expression as potential biomarkers.

## 2 MATERIALS AND METHODS

### 2.1 Cell lines, medium and reagents

Human colorectal cancer cell lines, including HCT116, SW480, Lim1215, RKO, DLD1 and HT29, were obtained from American Type Culture Collection (ATCC). The cell lines were grown in modified media McCoy's 5A (Invitrogen) in a 37°C incubator with 5% CO_2_ atmosphere, supplemented with FBS (10%; HyClone), streptomycin (100 μg/mL) and penicillin (100 units/mL), both from Invitrogen.

Cells were grown in 12‐well plates and at 20%‐30% density and were treated with the drugs. The antitumour agents used in this study including: gilteritinib from Active Biochem, 5‐fluorouracil (5‐FU), BAY 11‐7082 from Merck Chemicals, cisplatin (ImClone), and quizartinib and dovitinib from Selleckchem. The dilutions of all agents were dissolute in DMSO (Sigma‐Aldrich), excluding cisplatin, which was solubilized in NaCl (0.9%). One hour prior to gilteritinib treatment, BAY 11‐7082 was added to cells to test for NF‐κB inhibition.

### 2.2 PUMA knockout via CRISPR‐Cas9

The PUMA knockout cells were generated by selecting two gRNA sequences (GCTCCCCGGAGCCCGTAGAG and GTAGAGGGCCTGGCCCGCGA) that target PUMA and the synthesis of complementary, single‐stranded oligos having BsmBI overhangs were done. The lentiviral vector LentiCRISPR v2 from Addgene was cleaved using BsmBI FastDigest (Fermentas) and the clean, cleaved product was obtained using the QIAquick Gel Extraction Kit (Qiagen), and elution in buffer EB. The oligos were phosphorylated and annealed in T4 ligation Buffer using T4 polynucleotide kinase from NEB by incubating at 37°C for 30 minutes, then at 90°C for 5 minutes, and cooled finally to 25°C (at 5°C/min). Then, the oligos to be annealed were mixed with the digested LentiCRISPR v2 vector, using the Quick Ligase enzyme in Quick Ligase Buffer, and then transformed into Stbl3 bacteria. Twenty‐four hours before transfection, human 293T cells at 2 × 10^6^ cells/plate were seeded on 60 mm tissue culture plates. Then, in serum‐free media, lentiviral product (1 µg) was mixed with plasmids pMD2G and psPAX, and the PolyJet agent and incubated for 15 minutes at room temperature. The mixture was added slowly to the cells, and after 2 days of transfection, the medium with lentiviral particles was obtained. To carry out lentivirus infection, seeding of 6‐well plates with HCT116 cells (4‐5 × 10^4^/well) was done and the infected cells were selected in puromycin (2 µg/mL) following 1 day of infection and maintained further at 37°C in an atmosphere with 5% CO_2_. The cells that survived were then seeded on a 96‐well plate to select for a single clone. The knockout cells were further confirmed by Western blotting.

### 2.3 MTS assay

The specified cell lines were seeded at 1 × 10^4^ cells/well density in 96‐well plates. After incubating overnight, gilteritinib was added to the wells at different concentrations and kept further for 72 hours. Next, using the MTS assay kit from Promega (Durham) the MTS (3‐(4,5‐dimethylthiazol‐2‐yl)‐5‐(3‐carboxymethoxyphenyl)‐2‐(4‐sulfophenyl)‐2H‐tetrazolium) assay was carried out as per instructions. Luminescence was determined using Perkin Elmer's Wallac Victor 1420 Multilabel Counter. Each experiment was carried out thrice in triplicate.

### 2.4 Assay to determine Apoptosis

Staining of nuclear was done using Hoechst 33258 from Invitrogen as described previously.^32^, ^33^ Annexin V/PI (propidium iodide) staining was carried out using annexin‐Alexa 488 (Invitrogen) and PI as per instructions. After differential centrifugation to isolate mitochondrial and cytosolic fractions and the release of cytochrome c was assessed through Western blotting.

### 2.5 Colony formation

Colony formation was assayed by plating the treated cells in 6‐well plates at appropriate dilutions, and, grown for 2 weeks, after which, they were crystal violet from Sigma‐Aldrich stained. The changes in membrane potential of the mitochondria were evaluated flow cytometrically by staining the treated cells with MitoTracker Red CMXRos from Invitrogen at room temperature for 15 minutes.

### 2.6 Western blotting

Western blotting was performed as previously described.^34^, ^35^ Briefly, Western blotting was done using cell lysate (40 μg) and tumour tissue homogenate was mixed with Laemmli buffer (BioRad) on Precast Gels Mini‐PRTOEAN^®^ TGX™ (BioRad). After electrophoresis, the separated proteins on gel were shifted to PVDF membranes and immunoblotted using primary antibodies against cleaved caspase 3 (#9661), cleaved caspase 9 (#9509), PUMA (#24633), Bim (#2819), Noxa (#14766), Bad (#9268), Bid (#8762), Bcl‐2 (#15071) (Cell Signaling Technology), Bcl‐xL (ab32370), cytochrome c (ab133504), Cox IV (ab14744), p73 (ab40658), p‐Foxo3a (ab47285), Foxo3a (ab109629), p‐p65 (ab86299), p65 (ab16502), AKT (ab8805), p‐AKT (ab81283), GSK‐3β (ab93926), p‐GSK‐3β (ab131097), V5 (ab9137) (Abcam), p‐STAT1 (sc‐81522), STAT1 (sc‐73070), Lamin A/C (sc‐7293) and β‐Actin (sc‐58673) (Santa Cruz Biotechnology). After the blots were incubated with anti‐rabbit/mouse IgG coupled with peroxidase provided by Cell Signaling Technology for 1 hour at room temperature, ECL was done to detect protein bands using Imaging System.

### 2.7 Real‐time reverse transcription PCR

The Mini RNA Isolation II Kit from Zymo Research was used to extract total RNA from untreated and drug‐treated cells as per the previous protocol.^36^, ^37^ To generate complementary DNA, total RNA (2 μg) and SuperScript III reverse transcriptase from Invitrogen were used. The primers used for PUMA were as follows: Forward: 5′‐CGACCTCAACGCACAGTACGA‐3′, and Reverse: 5′‐AGGCACCTAATTGGGCTCCAT‐3′; β‐Actin: Forward: 5′‐GACCTCACAGACTACCTCAT‐3′, and Reverse: 5′‐AGACAGCACTGTGTTGGCTA‐3′.

### 2.8 Assays for transfection and siRNA knockdown

Transfection of cells was done using lipofectamine 2000 from Invitrogen as per instructions. Experiments with the knockdown were performed using siRNA (200 pmole) 24 hours before treating with gilteritinib. The siRNA for human p65 (sc‐29410), GSK‐3β (sc‐35527), and p53 (sc‐29435) and control scrambled siRNA were procured from Santa Cruz Biotechnology.

### 2.9 NF‐κB nuclear translocation analysis

Pre‐treatment of HCT116 cells was done either with BAY11‐7082 or transfected with GSK‐3β siRNA, after which, gilteritinib treatment was done for 4 hours. The nuclear translocation of NF‐κB was analysed through immunofluorescence and nuclear fractionation. To carry out nuclear fractionation, cells were treated in 75 cm^2^ flasks and nuclear extracts were isolated using the NE‐PER cytoplasmic/nuclear extraction kit from Thermo Fisher as per instructions and examined by Western blotting for p65.

### 2.10 Reporter assay

Constructs of luciferase reporter in pBV‐Luc vector containing mutant or WT PUMA promoter sequence were made as described.^8^, ^37^ To determine reporter activities, cells were transfected with the reporters with WT or mutant PUMA, in addition to pCMVβ (Promega) used as the transfection control harbouring β galactosidase reporter. Luciferase activity was measured and normalized with samples that were transfected similarly but with no drug treatment. All reporter assays were carried out thrice, and in triplicate.

### 2.11 Chromatin immunoprecipitation (ChIP) assay

ChIP assay using p65 antibody was performed using the ChIP Assay Kit from Abcam as described.^38^ For evaluation, the precipitates were used to PCR amplify PUMA promoter fragment having putative κB sites by using primers 5′‐GTCGGTCTGTGTACGCATCG‐3′ and 5′‐ CCCGCGTGACGCTACGGCCC‐3′.

### 2.12 Xenograft tumours

The Institutional Animal Care and Use Committee at the Second Affiliated Hospital of Dalian Medical University approved all the animal experiments. The xenografts of WT and *PUMA*‐KO HCT116 were confirmed and evaluated as described. Briefly, 5 × 10^6^ cells were inoculated on both flanks of female athymic nude mice (age: 4‐6 weeks old) and tumours were established for 7 days. Then, for 10 consecutive days, mice were treated with 5 mg/kg/d gilteritinib (diluted in 10% DMSO), or by oral gavage for vehicle control. Mice were randomly grouped such that the average volume of the tumours was the same across the groups prior to treatment. The volumes of tumours were measured in 2 dimensions using a vernier caliper every other and expressed as mm^3^ using the formula ½ × length × width.^2^ After 5 days post‐treatment, mice were killed for Western blot analysis. Dissection of tumours was done and fixed in formalin (10%) and paraffin‐embedded. On the paraffin‐embedded tumour sections (5 μm), immunoassay for cleaved caspase 3 was performed with the secondary antibody conjugated with Alexa Fluor 488 from Invitrogen.

### 2.13 Statistical analysis

For statistical analyses, GraphPad Prism was used. *P* values were calculated by the Student's *t* test (*P* < .05 was reviewed as significant), and one‐way analysis of variance (ANOVA) was used for multiple groups. The means ± SD are displayed in the figures.

## 3 RESULTS

### 3.1 Gilteritinib promotes growth inhibition and induces apoptosis in CRC cells

**Figure 1:**
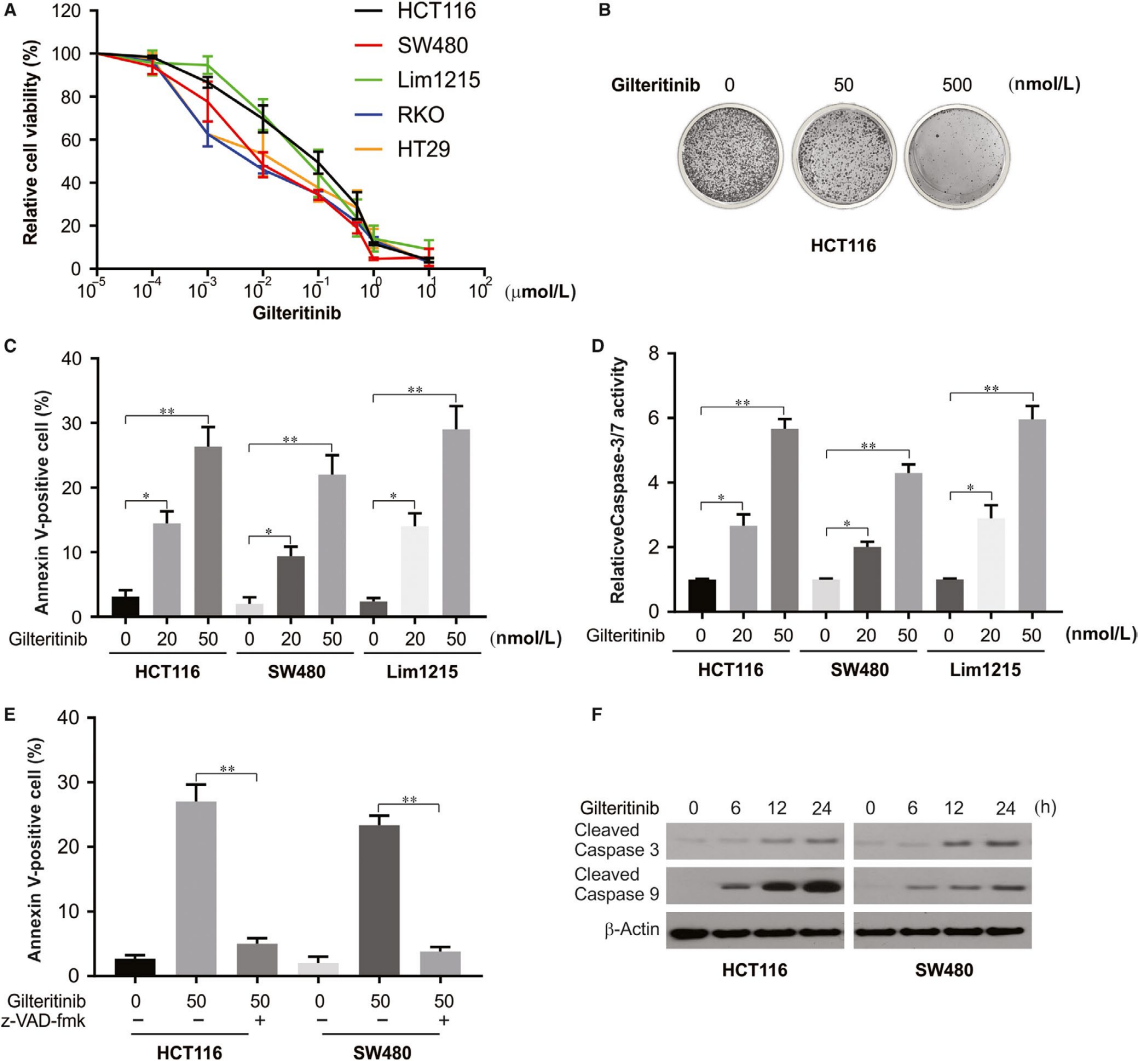
Gilteritinib inhibits cell growth and induces apoptosis in CRC cells. A, The indicated CRC cell lines were treated with increasing concentrations of gilteritinib for 72 h. Cell viability was determined by MTS assay. B, Crystal violet results of HCT116 cells treated with gilteritinib at indicated concentration for 72 h. C, The indicated cell lines were treated with gilteritinib for 24 h at indicated concentrations. Apoptosis was analysed by Annexin V/PI staining followed by flow cytometry. D, The indicated cell lines were treated gilteritinib at indicated concentration. Caspase 3/7 activity was determined by fluorogenic analysis. E, The indicated cell lines were treated 50 nmol/L gilteritinib with or without 10 μmol/L z‐VAD‐fmk pre‐treatment. Apoptosis was analysed by Annexin V/PI staining followed by flow cytometry. F, HCT116 and SW480 cells were treated with 50 nmol/L gilteritinib at indicated time‐point. Cleaved caspase 3 and 9 were analysed by Western blotting. Results in (C), (D) and (E) were expressed as means ± SD of three independent experiments. **P < .01; *P < .05

The CRC cell lines were treated with increasing concentrations of gilteritinib for 72 hours to assess the effects of gilteritinib on CRC, and MTS assay was performed to examine the cell growth inhibition. MTS results show that gilteritinib appreciably decreased viability in CRC cell lines (Figure 1A). Gilteritinib‐induced growth inhibition was further confirmed by crystal violet (Figure 1B). We next examined the mechanism of growth inhibition through the following experiments. We observed that gilteritinib increased the Annexin V‐positive populations of SW480, HCT116 and Lim1215 cells through apoptotic assays by flow cytometry (Figure 1C). Furthermore, our results also showed that gilteritinib increases caspases 3/7 activation in HCT116, SW480 and Lim1215 CRC cells in a dose‐dependent manner (Figure 1D). The apoptosis was shown to be blocked in SW480 and HCT116 cells pre‐treating with z‐VAD‐fmk, a pan‐caspase inhibitor (Figure 1E), implicating that caspase‐dependent apoptosis was induced by gilteritinib. Gilteritinib treatment in HCT116 and SW480 cells also promoted caspase 3 and 9 activation (Figure 1F). These findings indicate that gilteritinib induces cell growth inhibition and caspase‐dependent apoptosis in CRC cells.

### 3.2 Gilteritinib induces PUMA in CRC cells

**Figure 2:**
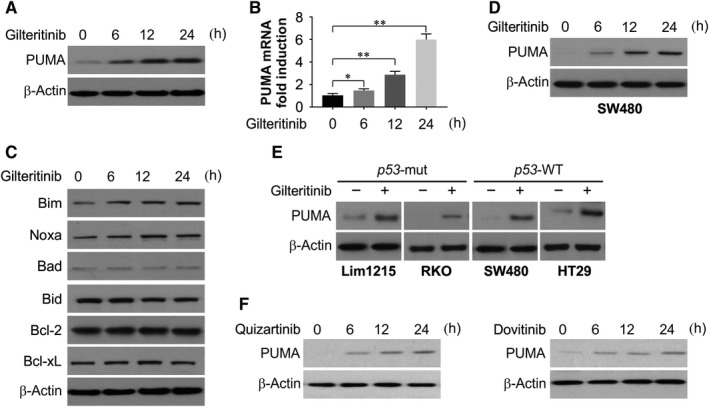
Gilteritinib promotes PUMA induction in CRC cells. A, HCT116 cells were treated with 50 nmol/L gilteritinib at indicated time‐point. PUMA expression was analysed by Western blotting. B, HCT116 cells were treated with 50 nmol/L gilteritinib at indicated time‐point. PUMA mRNA induction by gilteritinib was analysed by real‐time reverse transcriptase (RT) PCR. C, HCT116 cells were treated with 50 nmol/L gilteritinib at indicated time‐point. The expression of indicated Bcl‐2 family members was analysed by Western blotting. D, SW480 cells were treated with 50 nmol/L gilteritinib at indicated time‐point. PUMA expression was analysed by Western blotting. E, Indicated CRC cell lines were treated with 50 nmol/L gilteritinib for 24 hours. PUMA expression was analysed by Western blotting. F, HCT116 cells were treated with 50 nmol/L quizartinib or dovitinib at indicated time‐point. PUMA expression was analysed by Western blotting. Results in (B) were expressed as means ± SD of 3 independent experiments. **P < .01; *P < .05

The effects of gilteritinib on HCT116 CRC cells were studied to assess its effect on the expression of Bcl‐2 family proteins. Within 6 hours post‐treatment, PUMA protein and mRNA were induced suggesting regulation at transcriptional level (Figure 2A,B). Interestingly, there was an increase in expression of members of Bcl‐2 family, such as Bim and Noxa, while the levels of Bid, Bad, Bcl‐xL and Bcl‐2 were unchanged until 24 hours (Figure 2C). Induction of PUMA was observed to be stimulated time‐dependently by gilteritinib in SW480 cells (Figure 2D). This was also observed in other CRC cell lines, like *p53*‐mutant DLD1 and HT29 cells and in *p53*‐WT Lim1215 cells (Figure 2E). In addition, other FLT3 inhibitors, such as quizartinib and dovitinib, also induce PUMA up‐regulation in HCT116 cells (Figure 2F). Our results indicate that gilteritinib induces PUMA up‐regulation in CRC cells via a *p53*‐independent manner.

### 3.3 PUMA is required for gilteritinib‐induced apoptosis

**Figure 3:**
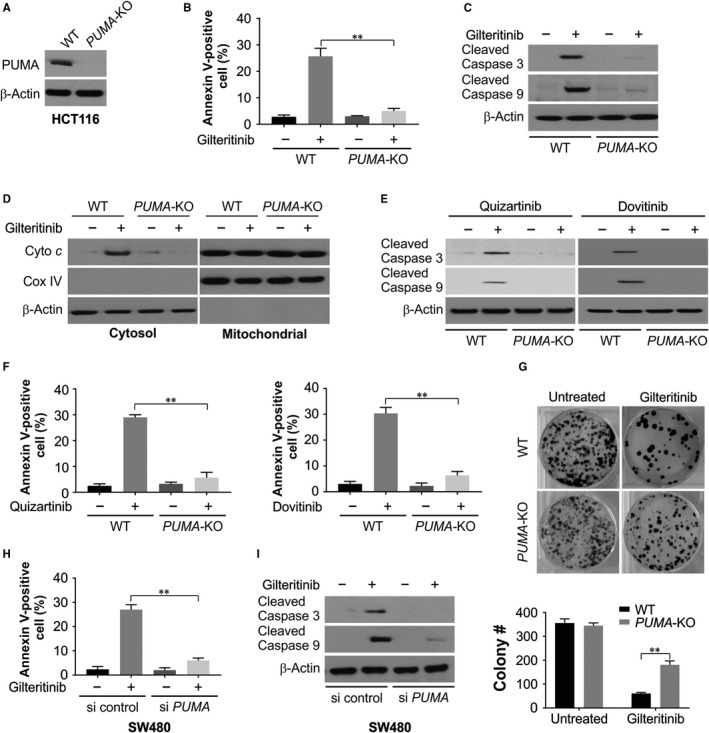
PUMA is required for the apoptotic and antitumour effects of gilteritinib via mitochondrial pathway. A, PUMA expression was analysed by Western blotting in WT and *PUMA*‐KO HCT116 cells. B, WT and *PUMA*‐KO HCT116 cells were treated with 50 nmol/L gilteritinib for 24 h. Apoptosis was analysed by Annexin V/PI staining followed by flow cytometry. C, WT and *PUMA*‐KO HCT116 cells were treated with 50 nmol/L gilteritinib for 24 h. Cleaved caspase 3 and 9 were analysed by Western blotting. D, Cytosolic fractions isolated from WT and *PUMA*‐KO HCT116 cells treated with 50 nmol/L gilteritinib for 24 h were probed for cytochrome c by Western blotting. β‐Actin and cytochrome oxidase subunit IV (Cox IV), which are expressed in cytoplasm and mitochondria, respectively, were analysed as the control for loading and fractionation. E, WT and *PUMA*‐KO HCT116 cells were treated with 50 nmol/L quizartinib or dovitinib for 24 h. Cleaved caspase 3 and 9 were analysed by Western blotting. F, WT and *PUMA*‐KO HCT116 cells were treated with 50 nmol/L quizartinib or dovitinib for 24 h. Apoptosis was analysed by Annexin V/PI staining followed by flow cytometry. G, Colony formation of WT and *PUMA*‐KO HCT116 cells treated with 50 nmol/L gilteritinib for 24 h at 10 d following crystal violet staining of attached cells. Upper, representative pictures of colonies; Bottom, quantification of colony numbers. H, SW480 cells transfected with si control or si *PUMA* were treated with 50 nmol/L gilteritinib for 24 h. Apoptosis was analysed by Annexin V/PI staining followed by flow cytometry. I, SW480 cells transfected with si control or si *PUMA* were treated with 50 nmol/L gilteritinib for 24 h. Cleaved caspase 3 and 9 were analysed by Western blotting. Results in (B), (F), (G) and (H) were expressed as means ± SD of 3 independent experiments. **P < .01

To assess the role of PUMA, we used CRISPR‐Cas9 system to generate PUMA knockout (*PUMA*‐KO) in HCT116 cells (Figure 3A). Treatment with gilteritinib induced apoptosis, cleaved caspase 3 and 9 and released cytochrome c in WT HCT116 cells, which was occluded in *PUMA*‐KO cells (Figure 3B‐D). In addition, *PUMA*‐KO cells are resistant to quizartinib‐ and dovitinib‐induced apoptosis and caspase 3 and 9 cleavage. (Figure 3E,F). In agreement with blocked apoptosis, we observed that the clonogenic survival was improved notably in *PUMA*‐KO cells (Figure 3G). On transiently knocking down PUMA using siRNA, and after gilteritinib treatment, we observed decrease in apoptosis and activation of caspases 3 and 9 in SW480 cells (Figure 3H,I). Thus, PUMA and the mitochondrial pathway play a vital role in gilteritinib‐induced apoptosis in CRC cells.

### 3.4 Gilteritinib‐induced PUMA expression is mediated by NF‐κB

**Figure 4:**
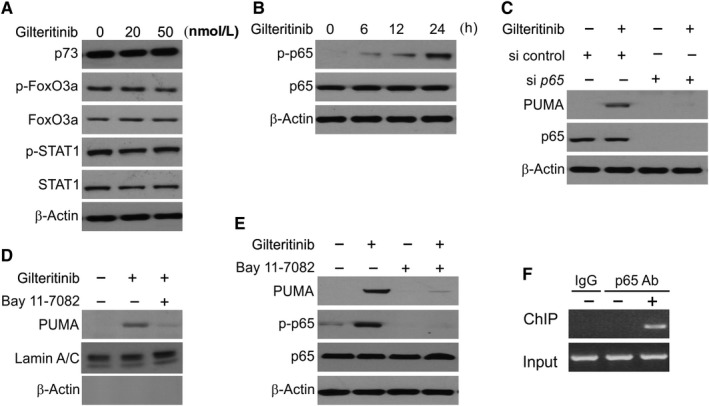
Activation of p65 mediates PUMA induction in response to gilteritinib treatment. A, HCT116 cells were treated with gilteritinib for 24 h at indicated concentration. Indicated proteins were analysed by Western blotting. B, HCT116 cells were treated with 50 nmol/L gilteritinib at indicated time‐points. Expression of p‐p65 (S536) and p65 was analysed by Western blotting. C, HCT116 cells were transfected with either a control scrambled siRNA or a p65 siRNA for 24 h and then treated with 50 nmol/L gilteritinib for 24 h. p65 and PUMA expressions were analysed by Western blotting. D, HCT116 cells were treated with 10 μmol/L BAY11‐7082 for 1 h, and then with 50 nmol/L gilteritinib for 24 h. Nuclear fractions were isolated from cells and analysed for p65 expression by Western blotting. Lamin A/C and β‐actin, which are expressed in nucleus and cytoplasm, respectively, were used as controls for loading and fractionation. E, HCT116 cells were treated with 10 μmol/L BAY11‐7082 for 1 h, and then with 50 nmol/L gilteritinib for 24 h. The level of p‐p65 (S536) and PUMA was analysed by Western blotting. F, Chromatin immunoprecipitation (ChIP) was performed using anti‐p65 antibody on HCT116 cells following gilteritinib (50 nmol/L) treatment for 12 h. The IgG was used to control for antibody specificity. PCR was carried out using primers surrounding the p65 binding sites in the PUMA promoter

We then analysed the mechanism of *p53*‐independent induction of PUMA by gilteritinib in CRC cells in terms of several transcription factors. There was no change in inhibitory phosphorylation after gilteritinib treatment, revealing the non‐involvement of FoxO3a (Figure 4A). Other p53 family member, p73 and STAT1, which was seen to mediate the sorafenib effects in the cancer cells of pancreatic,^39^ were also excluded because they did not mediate induction or any change in phosphorylation (Figure 4A). Recently, the NF‐κB p65 subunit was discovered as a PUMA transcriptional activator as a result of treatment with TNF‐α or sorafenib.^39^, ^40^ NF‐κB signalling activation is characterized by p65 phosphorylation on several residues and followed by its nuclear translocation, where it activates target gene transcription.^41^

We also observed that treatment with gilteritinib in HCT116 cells induced S536 phosphorylation, a prominent regulatory site of p65 (Figure 4B). p65 knockdown in HCT116 cells by siRNA overruled induction of PUMA by gilteritinib (Figure 4C). Furthermore, treatment with gilteritinib resulted in p65 nuclear translocation as observed in Western blot assay (Figure 4D). The mechanism of NF‐κB activation of PUMA transcription as a result of gilteritinib treatment was studied by pre‐treating the cells with BAY 11‐7082, an inhibitor of NF‐κB that represses nuclear translocation of p65 (Figure 4D). Treatment with BAY 11‐7082 led to gilteritinib‐induced impediment of PUMA expression and phosphorylation of p65 (Figure 4E), indicating that induction of PUMA by gilteritinib occurs via nuclear translocation of p65. We next determined through ChIP assay any direct role of NF‐κB in activating PUMA transcription and found that after gilteritinib treatment, p65 gets recruited to the PUMA promoter containing κB sites (Figure 4F). Thus, p65 binds directly to the PUMA promoter to steer its transcriptional activation as a result of gilteritinib treatment.

### 3.5 PUMA is induced by activated GSK3β after gilteritinib treatment

**Figure 5:**
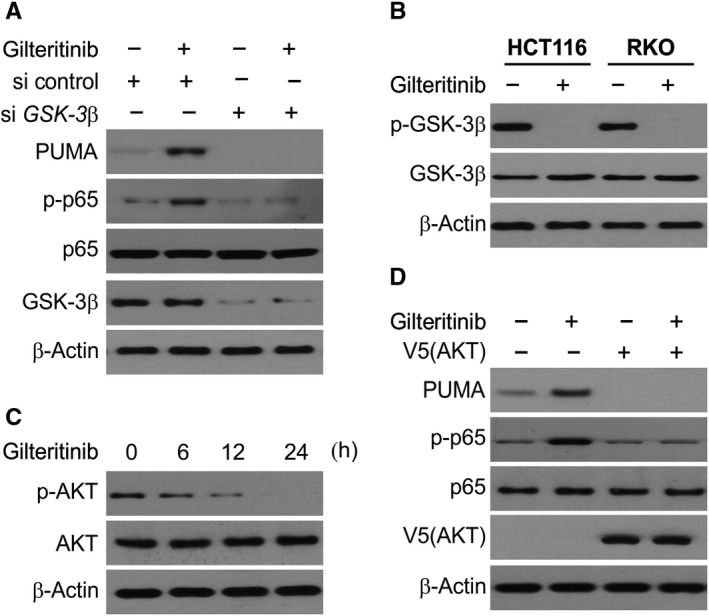
Gilteritinib activates GSK3β and inhibits AKT to induce PUMA through p65 activation. A, HCT116 cells were transfected with either a control scrambled siRNA or a *GSK3β* siRNA for 24 h, and then treated with 50 nmol/L gilteritinib for 24 h. Indicated proteins were analysed by Western blotting. B, HCT116 and RKO cells treated with 50 nmol/L gilteritinib for 24 h. The levels of total GSK3β and p‐GSK3β (S9) were analysed by Western blotting. C, HCT116 cells treated with 50 nmol/L gilteritinib at indicated time‐points. The levels of total AKT and p‐AKT were analysed by Western blotting. D, HCT116 cells transfected with AKT were treated with 50 nmol/L gilteritinib for 24 h. Indicated proteins were analysed by Western blotting

We assessed the involvement of GSK3β in gilteritinib‐induced p65 activation. We observed that *GSK3β* siRNA suppressed gilteritinib‐induced p65 phosphorylation, an effect not seen by the control siRNA (Figure 5A). The depletion of GSK3β in HCT116 cells also nullified induction of PUMA through gilteritinib (Figure 5A). The results also implicate that GSK3β gets dephosphorylated (Ser9) and subsequently inactivated after gilteritinib treatment, in HCT116 and *p53*‐mutant RKO cells (Figure 5B). We have seen that AKT phosphorylates Ser9 of GSK3β and inhibits its activity. In turn, gilteritinib treatment significantly suppressed AKT activation (Ser473), with no effect on their total levels (Figure 5C). Moreover, when the constitutively active AKT was overexpressed, it led to repression of PUMA induction and activation of p65 through gilteritinib (Figure 5D). The above results indicate that GSK3β is activated after AKT inhibition and leads to translocation of p65 and induction of PUMA by gilteritinib.

### 3.6 PUMA mediates the chemosensitizing effects of gilteritinib

**Figure 6:**
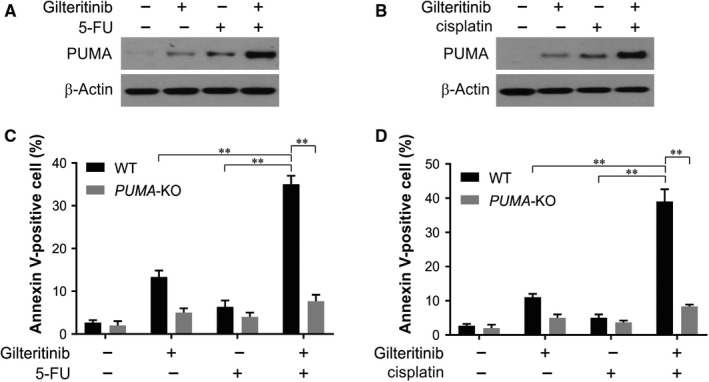
PUMA mediates the chemosensitization effects of gilteritinib. A, HCT116 cells were treated with 20 nmol/L gilteritinib, 20 mg/L 5fluorouracil (5‐FU) or their combination. PUMA expression was analysed by Western blotting. B, HCT116 cells were treated with 20 nmol/L gilteritinib, 25 mg/L cisplatin or their combination. PUMA expression was analysed by Western blotting. C, WT and *PUMA*‐KO HCT116 cells were treated with 20 nmol/L gilteritinib, 20 mg/L 5fluorouracil (5‐FU) or their combination. Apoptosis was analysed by Annexin V/PI staining followed by flow cytometry. D, WT and *PUMA*‐KO HCT116 cells were treated with 20 nmol/L gilteritinib, 25 mg/L cisplatin or their combination. Apoptosis was analysed by Annexin V/PI staining followed by flow cytometry. Results in (C) and (D) were expressed as means ± SD of 3 independent experiments. **P < .01

Next, we checked whether the simultaneous induction of PUMA by gilteritinib and other agents via different pathways resulted in chemosensitization. We observed that a notably higher level of PUMA was induced by gilteritinib in combination with 5‐FU or cisplatin than single treatment (Figure 6A,B). This is consistent with the simultaneous induction of PUMA through *p53*‐dependent, as well as ‐independent process by DNA damage and gilteritinib, respectively. Likewise, following the combination treatment, the level of apoptosis seen to increase significantly in WT HCT116 cells, but not in *PUMA*‐KO cells (Figure 6C,D).

### 3.7 The antitumour activity of gilteritinib in vivo requires PUMA

**Figure 7:**
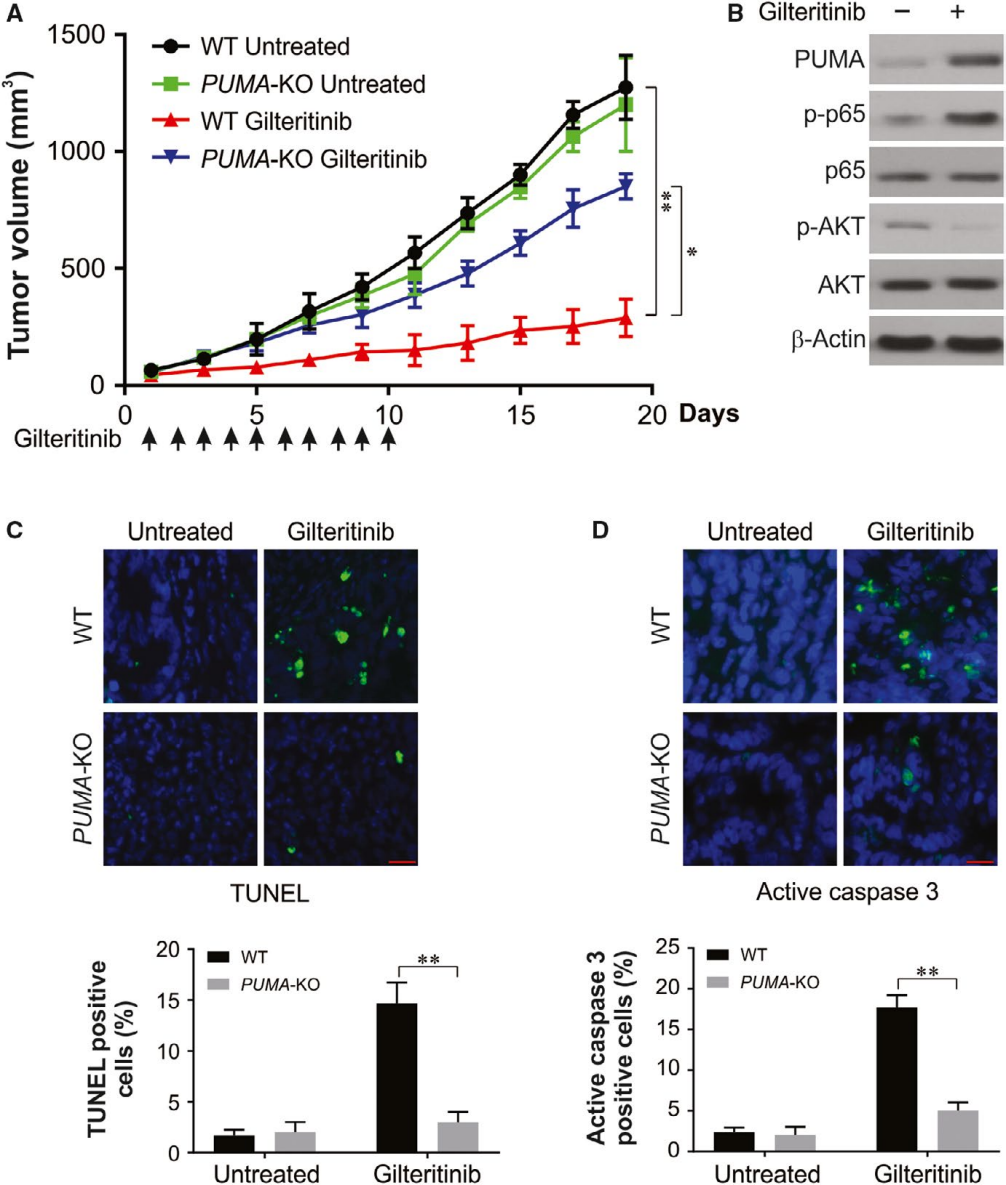
PUMA mediates the antitumour effects of gilteritinib in vivo. A, Nude mice were injected s.c. with 5 × 10^6^ WT or *PUMA*‐KO HCT116 cells. After 1 wk, mice were oral gavaged with 5 mg/kg gilteritinib or the vehicle control for 10 consecutive days. Tumour volume at indicated time‐points after treatment was calculated and plotted with *P* values, n = 6 in each group. Arrows indicate gilteritinib injection. B, Mice with WT HCT116 xenograft tumours were treated with 5 mg/kg gilteritinib or the vehicle for 5 consecutive days. The levels of indicated proteins in randomly selected tumours were analysed by Western blotting. C, Paraffin‐embedded sections of WT or *PUMA*‐KO tumour tissues from mice treated as in (B) were analysed by TUNEL staining. D, Paraffin‐embedded sections of WT or *PUMA*‐KO tumour tissues from mice treated as in (B) were analysed by activated caspase 3 staining. Results in (C) and (D) were expressed as means ± SD of three independent experiments. **P < .01

The PUMA‐mediated tumour suppression by gilteritinib was examined by injecting nude mice subcutaneously with WT and *PUMA*‐KO HCT116 cells to establish xenograft tumours and was then treated with 5 mg/kg gilteritinib or the control vehicle and the tumours were observed. In the control group, there was no significant difference in growth of WT and *PUMA*‐KO tumours (Figure 7A). While the WT tumour growth was suppressed by 70%‐80% upon gilteritinib treatment (Figure 7A), *PUMA*‐KO tumours were significantly less sensitive to the treatment (Figure 7A), indicating that the antitumour activity of gilteritinib is nullified in the absence of PUMA. Consistently, p65 phosphorylation and expression of PUMA were markedly enhanced and AKT phosphorylation was reduced in xenograft tumours treated with gilteritinib (Figure 7B). On TUNEL staining, significant induction in apoptosis was seen in WT tumour tissues in mice treated with gilteritinib, but not in control mice. In contrast, the *PUMA*‐KO tumours exhibited largely reduced apoptosis (Figure 7C). Staining for cleaved caspase 3 further confirmed apoptosis in tumours induced by gilteritinib and dependent on PUMA (Figure 7D). Hence, the in vivo antitumour and apoptotic activity of gilteritinib rely substantially on PUMA.

## 4 DISCUSSION

Although one of the promising drug targets is aberrantly activated oncogenic kinases,^42^ biomarkers, the resistance mechanisms and potential of most clinically useful kinase inhibitors remain majorly unexploited. Gilteritinib inhibits FLT3 with high specificity and potency, and shows antileukaemic activity against FLT3‐ITD mutations in the presence or absence of TKD mutations.^20^ Gilteritinib displayed clinical activity across a wide therapeutic window and was well tolerated and in a population of heavily pre‐treated FLT3^mut+^ R/R AML.^19^ Gilteritinib, a small molecule, is an inhibitor of this pathway and is FDA (Food and Drug Administration) approved for treating AML.^22^ This is the first study to demonstrate that tumour suppressor activity of gilteritinib is dependent on the autonomous apoptotic induction, beginning from inhibition of AKT, activation of GSK3β and nuclear translocation of p65, resulting in induction of PUMA and initiation of mitochondria‐mediated apoptosis. Moreover, the gilteritinib and 5‐FU or cisplatin combinations lead to robust induction of apoptosis through PUMA in CRC cells.

The induction of PUMA has a vital role in apoptosis induced by a range of chemotherapy agents and may be a valuable chemosensitivity biomarker.^37^ Studies have shown that induction of PUMA relates closely with varying sensitivity to EGFR TKIs in neck and head cancer cells, and of the absence of PUMA induction correlates with resistance to EGFR TKIs.^8^, ^43^ Increased expression of PUMA is associated with superior prognosis in stage II and III CRC patients undergoing 5‐FU‐based therapy.^44^ Therefore, induction of PUMA may be a useful surrogate biomarker for CRC response to gilteritinib. While obtaining biopsies from colorectal tumours treated with chemotherapy is often difficult, recent studies used circulating tumour DNA, or cells, and successfully analysed the biomarkers of therapeutic response.^45^ Thus, the analysis of PUMA induction by using such non‐invasive approaches is a good possibility.

The therapeutic efficacy of combining gilteritinib and 5‐FU may be responsible for induction of PUMA through two distinct mechanisms. Besides, *p53*‐dependent and/or ‐independent PUMA induction also intercedes apoptotic response to other targets, such as the drug sunitinib, a multi‐kinase inhibitor,^46^ and 17‐AAG, an Hsp90 inhibitor.^47^ As an effective indicator of drug combinations, induction of PUMA may be useful in developing more efficient combination regimens with decreased doses and non‐overlapping toxicities.^38^

Chemotherapy is majorly limited through drug resistance, especially in the case of targeted therapies.^48^ Assessing the effectiveness of PUMA‐mediated apoptosis can be used to unravel potent antitumour agents that surpass chemoresistance. Recent years have seen the development of a number of apoptosis‐targeting agents in various clinical trials.^49^, ^50^ These agents that target apoptosis may act synergistically with gilteritinib, one of such chemotherapeutic drugs. While the results of this study were encouraging, our future investigations will include further clinical relevance on other pre‐clinical models and human patient specimens from clinical trials.

In summary, this is the first report in which the antitumour mechanism of gilteritinib was demonstrated through apoptosis mediated by PUMA. Up‐regulation of gilteritinib‐induced PUMA has the potential to act as a biomarker for the clinical studies and provides valuable implications for drug development and therapy.

## CONFLICT OF INTEREST

The authors declare no conflict of interest.

## AUTHOR’S CONTRIBUTION

LJL and LL designed the research study. LJL, ML and WLL performed the research. LL analysed the data. LJL wrote the paper and submitted the manuscript.

## Data Availability

All data generated or analysed during this study are included in this published article. And all data used to support the findings of this study are available from the corresponding author upon request.
